# Supplementary material for: Antagonistic Efficacy of Luteolin against Lead Acetate Exposure-Associated with Hepatotoxicity is Mediated via Antioxidant, Anti-Inflammatory, and Anti-Apoptotic Activities
Source: Antioxidants (Basel). 2019 Dec 21;9(1):10. doi: 10.3390/antiox9010010 (PMC7022878; doi:10.3390/antiox9010010)
Supplement: Supplementary file 1 [file antioxidants-09-00010-s001.pdf]

Supplementary data Table S1. Effect of luteolin (LUT, 50 mg/kg, orally) and/or lead acetate (PbAc, 20 mg/kg, i.p.) exposure in body weight, and food and water intake of male rats.

**Groups**	**Body weight (g)**	**Food intake ($g\ day^{-1}$)**	**Water intake ($ml\ day^{-1}$)**
**CNTR**	180.2±9.81	21.3±2.5	28.9±2.2
**LUT**	182.8±10.13	20.9±1.7	29.3±1.9
**PbAc**	155.6±9.56[a]	17.1±0.8[a]	26.1±2.1[a]
**LUT+PbAc**	170.6±9.45[b]	20.1±1.6[ab]	28.2±2.4[b]

The values represent the means ± SD (n = 7). [a] represents the statistical significance relative to that of the control group at $p<0.05$. [b] represents the statistical significance relative to that of the PbAc-injected group at $p<0.05$.
